# Greening of the Heart and Mind

**DOI:** 10.1161/JAHA.119.012090

**Published:** 2019-03-05

**Authors:** John R. Balmes

**Affiliations:** ^1^ Department of Medicine University of California San Francisco CA; ^2^ Division of Environmental Health Sciences School of Public Health University of California Berkeley CA

**Keywords:** Editorials, cardiovascular disease prevention, cardiovascular disease risk factors, environment, mental health, obesity, physical exercise, Cardiovascular Disease, Exercise, Mental Health, Risk Factors, Obesity

## Abstract

See Article by Wang et al

The impact of the built environment on health has been increasingly recognized by public health practitioners over the past 2 decades,^1^ yet this impact is not well appreciated by most clinicians. Perhaps this lack of appreciation is due to the fact that most of the evidence in support of built environment–health outcome relationships comes from observational epidemiological studies rather than randomized controlled trials, the sine qua non of evidence‐based medicine. However, when multiple epidemiological studies demonstrate associations between a risk or preventive factor and one or more health outcomes—that is, when there is consistency and coherence among studies—then clinicians should pay heed. The built environment is one such factor that can either positively or negatively affect cardiovascular outcomes.

The concept of the built environment involves multiple components, including neighborhood design (eg, walkability, bikeability, connectivity), housing quality, transportation facilities (eg, highways, railroads, ports, airports), power plants, industrial facilities, food accessibility, liquor and tobacco stores, crime, and green space. The presence or absence of these components helps determine the impact on health by increasing or decreasing psychosocial stress, exposure to air pollution and noise, and the ability to exercise safely and to eat a healthy diet.

Cities that developed before motor vehicles were introduced tend to be more densely populated and more walkable than newer cities, which tend to be less densely populated and more reliant on automobiles for transportation (ie, urban sprawl). Because of the mass use of automobiles, these newer cities often have inadequate public transportation, poor infrastructure for active commuting, lack of green space, and higher exposures to air pollution and noise. These conditions can lead to sedentary behavior, obesity, and high blood pressure and, subsequently, higher rates of cardiovascular morbidity and mortality.

Built environmental factors are modifiable, and interventions at the community level, such as urban planning, may have wider beneficial health impact than interventions designed to change behavior at the individual level. Because changes to the environment require policy decisions that have ramifications for other parts of complex urban systems, policy makers require better data on how environmental factors are linked to cardiovascular health outcomes.

One modifiable environmental factor for which the literature is relatively sparse is *green space*, defined as an area of grass, trees, or other vegetation set apart for recreational or aesthetic purposes in an otherwise urban environment.^2^ A meta‐analysis published in 2015 showed that the risk of cardiovascular disease mortality was statistically significantly reduced in 5 of 8 studies evaluating the association between risk and residential greenness.^3^ The reduced risks were relatively small in most of these studies, and the pooled reduced risk was 8%. Other studies not included in this meta‐analysis support a beneficial effect of greenness on cardiovascular disease mortality or morbidity. One such study in the United States found increased cardiovascular disease mortality in areas where there was a die‐off of trees due to an infestation.^4^ An Australian study demonstrated reduced risk of hospitalization for both cardiovascular and cerebrovascular disease in areas with greater residential greenness.^5^ A recent study from Hong Kong reported similar findings for cardiovascular disease mortality.^6^

The report by Brown et al in this issue of the *Journal of the American Heart Association (JAHA)* is an important contribution to the relatively limited existing literature on green space and cardiovascular disease.^7^ Using both a well‐validated greenness exposure database (the Normalized Difference Vegetation Index [NDVI]) and Medicare beneficiary data regarding acute myocardial infarction, ischemic heart disease, heart failure, and atrial fibrillation, the authors assessed the associations between neighborhood greenness and the prevalence of each cardiovascular disease outcome in Miami‐Dade County, Florida. They used hierarchical regression analyses in a multilevel framework to successively adjust for individual sociodemographics, neighborhood income, and biological risk factors (diabetes mellitus, hypertension, hyperlipidemia). As hypothesized, higher neighborhood greenness was associated with reduced risk of the 4 outcomes, and these results were robust with adjustment for individual sociodemographics and neighborhood income. The associations with greenness were attenuated somewhat with adjustment for biological risk factors, suggesting that these risk factors may be mediators of the associations.

Strengths of the study by Brown et al include census block–level assessment of greenness (NDVI) using a well‐validated database of satellite imagery, the large population‐based sample size (≈250 000), appropriate statistical analysis, and adjustment for relevant covariates. The authors acknowledge that the cross‐sectional nature of their analyses limits causal inference because healthier individuals may have elected to live in greener environments. Other limitations include lack of inclusion of (1) other environmental exposures besides residential block greenness such as air and noise pollution and (2) individual behavioral information such as smoking, diet, and physical activity that may have led to unmeasured confounding of the observed associations. Despite these limitations, this study makes an important contribution as the first study to show an association between higher levels of residential block greenness and lower odds of common heart diseases.

This observational epidemiological study cannot directly address potential mechanisms underlying the observed associations, but the attenuation of the odds with adjustment for diabetes mellitus, hypertension, and hyperlipidemia provide some clues. Green space may promote physical activity, but the literature contains conflicting evidence.^8^ That said, a large Australian study (n>200 000) found that higher green space as a percentage of the total land use within a 1‐km radius of a residence substantially increased the likelihood of moderate to vigorous physical activity.^9^ Potential effects of green space on obesity and blood pressure might be related to increased physical activity. Another potential mechanism by which green space can affect cardiovascular health is through stress reduction. A fairly robust literature supports green space as a protective factor for mental health.^10^

The measure of greenness exposure used by Brown et al, the NDVI, does not identify specific types of vegetation or greenery or whether the greenness was publicly accessible. Although large public parks are the classic type of green space, private areas of vegetation and smaller parks can also provide greenness to a developed area. Some evidence in the literature supports an exposure–response relationship for the effect of green space on stress reduction and mental health.^11^ A study from New York City reported that living in a neighborhood with small parks was associated with lower body mass index, as was living near large parks.^12^

What does all this mean for the practicing physician? The Centers for Disease Control and Prevention currently recommends 150 minutes a week of moderate‐intensity (or 75 minutes of high‐intensity) aerobic activity to improve the health of the general population.^13^ Physicians are encouraged to prescribe exercise for their patients, especially for those with risk factors for cardiovascular disease. The results of the study by Brown et al placed in context with the existing literature suggest that physicians recommend physical activity to occur in green spaces as much as possible.

A final message involves green space and climate change. Green space in high‐density urban areas reduces the heat island effect.^14^ Heat stress is another risk factor for acute morbidity among patients with preexisting cardiovascular disease, especially among elderly, socially isolated, and impoverished individuals who cannot afford air conditioning or get to cooling centers easily.^15^ Parks and urban forestry can reduce ambient temperature and thereby reduce risk of heat stress.^16^ Physicians can use the data in the scientific literature to advocate for green urban planning in their communities to improve the health of their patients.

## Disclosures

None.
